# Interactions between multiple helminths and the gut microbiota in wild rodents

**DOI:** 10.1098/rstb.2014.0295

**Published:** 2015-08-19

**Authors:** Jakub Kreisinger, Géraldine Bastien, Heidi C Hauffe, Julian Marchesi, Sarah E Perkins

**Affiliations:** 1 Department of Biodiversity and Molecular Ecology, Centre for Research and Innovation, Fondazione Edmund Mach, Via E. Mach 1, 38010 S. Michele all'Adige, TN, Italy; 2 Centre for Digestive and Gut Health, Department of Surgery and Cancer, Faculty of Medicine, Imperial College London, London SW7 2AZ, UK; 3 School of Biosciences, Cardiff University, Sir Martin Evans Building, Museum Avenue, Cardiff CF10 3AX, UK

**Keywords:** wildlife microbiota, community interactions, metagenome, infracommunity, *Apodemus flavicollis*

## Abstract

The gut microbiota is vital to host health and, as such, it is important to elucidate the mechanisms altering its composition and diversity. Intestinal helminths are host immunomodulators and have evolved both temporally and spatially in close association with the gut microbiota, resulting in potential mechanistic interplay. Host–helminth and host–microbiota interactions are comparatively well-examined, unlike microbiota–helminth relationships, which typically focus on experimental infection with a single helminth species in laboratory animals. Here, in addition to a review of the literature on helminth–microbiota interactions, we examined empirically the association between microbiota diversity and composition and natural infection of multiple helminth species in wild mice (*Apodemus flavicollis*), using 16S rRNA gene catalogues (metataxonomics). In general, helminth presence is linked with high microbiota diversity, which may confer health benefits to the host. Within our wild rodent system variation in the composition and abundance of gut microbial taxa associated with helminths was specific to each helminth species and occurred both up- and downstream of a given helminth's niche (gut position). The most pronounced helminth–microbiota association was between the presence of tapeworms in the small intestine and increased S24–7 (Bacteroidetes) family in the stomach. Helminths clearly have the potential to alter gut homeostasis. Free-living rodents with a diverse helminth community offer a useful model system that enables both correlative (this study) and manipulative inference to elucidate helminth–microbiota interactions.

## 1. Introduction

Mammals have coevolved with their gut microbial community (microbiota) for approximately 500 million years [1,2]. Recent advances in sequencing technology have begun to shed light on microbiota composition, structure and function, to discover the underlying mechanisms driving microbiota variation, and to identify the clinical implications of changes in these bacterial communities. This burgeoning field has led to the discovery that the gut microbiome (the combined genetic material of the microbiota) provides essential host services, from absorbing and generating vitamins, through regulating cognition and behaviour, to protection from pathogenic microbes, immune system development and the prevention of auto-immune disease [1,3,4].

A prevalent biotic component of the gut is parasitic worms, such as cestodes and nematodes (referred to here as ‘helminths’). Helminths are common in the gastrointestinal tracts of livestock, wildlife and humans and especially in pre-industrialized countries where millions of people are infected globally [5]. Given their long coevolutionary history and sympatric distribution inside the host it would be surprising if the gut helminths and microbiota did not interact [6,7]. Heavy infection is associated with economic loss through decrease in livestock production, and an increase in human morbidity, but at low levels of colonization, helminths usually cause asymptomatic or subclinical chronic infection [8,9]. The ability of helminths to be relatively benign may be explained by their suppression of host defence mechanisms. By producing a dominant T helper 2 (Th2) immune phenotype, among other effects, helminths can induce an anti-inflammatory environment, thereby redirecting immune responses away from themselves [8]. In fact, the results of experimental infections with helminths have led to the hypothesis that helminth-induced modulation of the immune system may be mediated via the host, or indirectly via changes in the microbiota [10], such that a three-way interaction between host, helminths and microbiota may occur (see [6] for a review). Whereas host–microbiota interactions are well studied, as are microbe–microbe interactions, especially concerning bacterial interference (e.g. [1,11]), empirical evidence for associations between microbiota and helminths is just beginning to emerge in the literature and is the focus of this paper.

Although competition between helminths for food resources [12], secretion of bacterial growth inhibitors by some species [13,14], and host age and diet [6,15] have all been proposed as mechanisms altering the gut microbiota, the interplay between host, helminths and microbiota has attracted much attention owing to the potential for helminths to induce direct or indirect changes in the microbiota, for example, via host immunity [6,16]. An absence of helminths or an incomplete microbiota community, known as biome depletion, within human populations has been widely cited as a mechanism leading to the increased prevalence of auto- and hyper-immune diseases [9,17]. As such, helminth modification of the gut microbiota may have the potential to be harnessed for valuable therapeutic approaches ([18,19] but see Rausch *et al.* [20]), hence the potential effects of helminths on host-microbiota composition and diversity deserve further investigation.

While the above observations suggest a three-way interaction between host, helminth and microbiota, to the best of our knowledge, only 10 studies to date have used a metataxonomic approach to investigate the association between gastrointestinal helminths and microbiota composition and diversity in mammals [10,16,18,20–26], with no broad consensus on the nature of the association. For example, changes in the composition of host microbiota owing to experimental infection with the nematode *Trichuris suis* has been observed in domesticated pigs [24], while experimental inoculation of laboratory mice with the common laboratory model nematode *Heligmosomoides polygyrus bakeri* has been associated with an increase in bacterial abundance at the site of helminth colonization, the ileum/small intestine [10], as well as within the caecum and colon [20]. By contrast, in humans, experimental removal of whipworm, *Trichurus trichuria* [22], or addition of hookworms, *Necator americanus* [21], both reported no change in host faecal microbiota diversity and composition. Experimental inoculation of helminths, however, can increase microbial diversity in individuals from which helminths are usually absent, for example laboratory animals [18].

As illustrated by the above review of microbiota–helminth associations within the literature, the gut ‘biome’ can be modulated by the interactions between host, microbiota and helminths [6]. The particular bacterial families present may shape these interactions. For example, the Lactobacillaceae family, in particular *Lacotobacillicus acidophilus* (lactic acid bacteria, LAB), has been proposed as key in bacterial interference; for example, inhibiting pathogen invasion in the honeybee [27]. Interestingly, LAB have been shown to significantly increase in abundance during colonization by *H. p. bakeri* in laboratory mice [10]. On the other hand, bacteria with metabolic potential are reduced in pigs infected with *T. suis*, such that these hosts cannot use carbohydrates fully [24]. Although the ‘benefit’ to the helminth in both of these examples is not clear, the interaction between helminths and microbiota does not appear to be unidirectional. In general, a gut microbiota appears to be required for helminth infection to occur [25,28]. In addition, probiotics have been used to control the proliferation of helminths and other eukaryotic organisms (see [29] for a review). Initial microbiota composition may also influence the development of parasitic infection within the gut [30], and in support of this hypothesis, Koch & Schmid-Hempel [31] provide empirical evidence that the microbiota phenotype and not hosts' genotype drives immune phenotypes, ultimately determining the nature of the host–parasite interaction.

It is important to note that not all interactions between helminths and microbes from the gut occur within the host. Evidence for interactions within the environment exists; infective free-living L3 helminth larvae of *Ostertagia ostertagi*, *Cooperia onchophora* and *Haemonchus contortus* have been found to harbour bacteria, probably acquired from the faecal microbiota of their hosts, cattle and sheep, in which they develop [32]. Interestingly, infective L3 *H. polygyrus* have been found to have a unique, but depauperate, microbiota community compared with the gut microbiota of their host niche [10]. Although microparasites can be introduced to the host via helminths [33], it remains to be shown whether helminths in natural ecosystems directly introduce a substantial and/or functional microbiota to the host. In addition, in laboratory mice, the successful maintenance of some helminth life cycles has been shown to be dependent on the host microbiota. More adult *H. polygyrus* nematodes, for example, develop in conventional versus germ-free mice [28]. In addition the size, fecundity and survival of the helminths are enhanced [34], while in conditions of gut dysbiosis unviable eggs are shed [35]. More recently, the hatching success of embryonated eggs of the common nematode *Trichuris muris* was found to require direct contact with five key bacterial strains and one yeast in order to match hatching rates of gut explants, providing evidence of a clear functional role of the host's microbiota [36]. Such interactions in wild populations of both hosts and helminths remain to be demonstrated, however.

As the above overview demonstrates, to date, helminth–microbiota interactions have primarily been addressed in laboratory and domesticated animals, using experimental inoculation [10,16,18,20,23–25,36,37], while studies in humans have been restricted to faecal analysis [21,22]. The high variation in microbiota composition along the gastrointestinal tract is such that the study of faecal microbiota does not quantify local changes at the sites of infection and data interpretation is considered limited [21]. No study has thus far examined ‘natural’ helminth–microbiota interactions in free-living populations, although Cooper *et al.* [22] examined the effect on microbiota of experimental removal of helminths in a cohort of naturally infected humans. The gut microbiota, gut helminths and immune responses in free-living mammals differs from that of laboratory and domesticated animals [38–40]. One key difference in free-living species is that multiple infections of simultaneously infecting parasites are the norm. The effect of multiple helminth infections has been little examined, but one study found that the faecal microbiota diversity of humans was reduced in mixed versus single infections [22]. Therefore, in order to understand the evolutionary basis of helminth–microbiota associations, it is crucial to explore natural systems, where microbial and helminth communities are intact. In addition, given potential complex interactions, we propose that a whole community approach to investigating interactions between the microbiota and multiple helminth species is needed. Here, we investigated the association between multiple helminth species and microbiota diversity, community composition and assumed function in multiple gut sections of a population of free-living wild yellow-necked mice, *Apodemus flavicollis*.

## 2. Material and methods

### (a) Wild rodent and gut microbiota collection

Nine female and 20 male adult *A. flavicollis* were live-trapped in September 2012 from three geographically distinct populations: Cadine (46°5′49.20″ N, 11°4′3.80″ E), Covelo (46°5′58.46″ N; 11°0′50.36″ E) and Pietramurata (46°1′5.14″ N; 10°56′15.05″ E) in the Province of Trento, Italy. Animals were euthanized *in situ* using isofluorane and stored at −80°C until dissection under sterile conditions. The entire gut was placed in TBS buffer (50 mM Tris, 200 mM NaCl, pH 8), and divided into stomach, small intestine, caecum and distal colon. For each of these sections, the luminal contents were centrifuged at 950*g* for 10 min at 4°C. The supernatant (luminal fluid) was centrifuged at 9000*g* for 15 min at 4°C and the resultant pellet (luminal bacteria) used for DNA extraction (see below). For each small intestine sample, the mucosa particles obtained from the first centrifugation were re-homogenized twice in TBS, centrifuged at 950*g* for 10 min at 4°C (hereafter ‘mucosa’). Each gut section was scanned under a Leica MS5 stereomicroscope (Leica Microsystems, Wetzlar, Germany) at 40× magnification to count the total number of each helminth per gut section per individual.

### (b) 16S rRNA gene sequencing

Total genomic DNA was extracted from each sample (luminal bacteria of stomach, small intestine, caecum and distal colon, and mucosa particles of small intestine) using the QIAamp DNA Stool Mini kit (Qiagen, Valencia, CA, USA). Methods followed the manufacturer's instructions for pathogen detection, with the addition of a 2 min homogenization step at 30 Hz to enhance bacterial cell lysis, using a Mixer Mill MM200 (Retsch GmbH, Haan, Germany) with 5 mm stainless steel beads (Qiagen). Purity and quality of the recovered DNA were determined using a Nanodrop 8000 (Thermo Scientific/Nanodrop, Wilmington, DE, USA) and a QIAxcel capillary electrophoresis system (Qiagen). The V1–V3 regions of the 16S rRNA gene were amplified with the primers 27F and 533R and pyrosequenced using the GS FLX + system (454 Life Sciences, Roche, Basel, Switzerland). The forward primer included the Lib-L primer A, the key sequence TCAG, the sample-specific Roche barcode multiplex identifier (MID), and the 27F forward primer sequence; the reverse primer contained the Lib-L primer B sequence and the 533R primer (454 Sequencing System-Guidelines for Amplicon Experimental Design, Roche, Branford, CT, USA). Polymerase chain reactions (PCRs) were carried out in triplicate (using the same MIDs for each sample, then pooled) using 25 µl reactions with 0.4 µM of each primer, 5–20 ng of template DNA, 2.5 µl of the FastStart reaction 10× buffer and 1.25 U of FastStart High Fidelity Polymerase and the amplification program provided for the GS FLX + system (Amplicon Library Preparation Manual, June 2013, Roche). Negative controls were included every 12 samples. The PCR products were analysed on a 2100 Bioanalyzer (Agilent Technologies, Santa Clara, CA, USA) and cleaned using the Agencourt AMPure XP system (Beckman Coulter, Brea, CA, USA) following the manufacturer's instructions. The products obtained were quantitated using the KAPA Library quantification kit for Roche 454 GS titanium platform (KAPA Biosystems, Boston, MA, USA) and pooled in an equimolar way in a final amplicon library. Pyrosequencing was carried out following the manufacturer's recommendations.

### (c) Bioinformatic processing of 16S data

Sff files were demultiplexed and quality filtered in QIIME (average *Q* > 25, sequence length > 200 bp, less than 3 ambiguous nucleotides, no mismatch in MID and F primer) following Caporaso *et al.* [41] and denoised using ACACIA [42]. USEARCH was used for identification and filtering of chimeric sequences and for de-novo clustering of resulting high-quality sequences into operational taxonomic units (OTUs) at a 97% similarity threshold [43], resulting in 5 629 700 high-quality reads (mean ± s.e. number of reads per sample = 40 605 ± 1962; range = 12 205–118 399). Taxonomic classification of representative sequences for individual OTUs was performed in RDB classifier [44], and a phylogenetic tree was constructed using FastTree [45] after PyNAST alignment [46]. We used PICRUSt [47] to predict the metagenome content of each sample (i.e. the bacterial community of each of the five gut sections for each *A. flavicollis* analysed). We mapped our high-quality sequences against the Green Gene reference OTUs (version gg_13_5_otus [48]; 91%, 93%, 95% and 97% similarity thresholds) using the closed reference algorithm implemented in QIIME. The proportion of sequences that were not assigned to any reference OTU was high at the 97%, 95% and 93% similarity thresholds (ranging from 14 to 39%) and relatively low in the case of 91% (7%). In addition, proportions of unassigned sequences varied between gut sections and the level of this variation was more pronounced at higher sequence identity thresholds. On the other hand, we observed only a slight increase of weighted Nearest Sequenced Taxon Index (NSTI, i.e. average branch length separating OTUs from a reference bacterial genome) with decreasing similarity threshold. NSTI for 97%–91% similarity threshold ranged between 0.126 and 0.164, which is comparable with data for other non-model mammalian species [47]. Therefore, to minimize the risk of bias owing to poor representation of our data in the reference database, we based the metagenomic predictions on a 91% similarity threshold as recommended by Langille *et al.* [47]. Nevertheless, the fact that the between-sample variation in predicted metagenome content was highly correlated across different similarity thresholds (range of Procrustes cor. coeff. = 0.99–0.93) is worth noting. The predicted metagenomes were classified according to Clusters of Orthologous Groups of proteins (COGs, [49]).

### (d) Statistical analyses

We describe here an overview of our general statistical approach to assess helminth–microbiota interactions. In each model, we examine helminth presence (a binary variable), abundance (defined as rank-transformed total number of helminths found in the rodent population, including zero values of uninfected hosts) and diversity (i.e. number of helminth species) unless otherwise stated. Standard indices, including microbiota diversity (number of OTUs), metagenome composition and OTU abundance were used as response variables (unless otherwise stated), and each of three common helminths (the nematodes *H. polygyrus* and *Syphacia* spp. and the cestode, *Hymenolepis* spp.) as explanatory variables. In all models, we considered the effect of geographical location to explicitly account for the assumed spatial variation of gut microbiota between populations, although this factor had a relatively low effect size in our dataset (data not shown). One helminth species, *Mastophorus muris,* was found in four animals from only one geographical location; therefore, to determine the relationship between the gut microbiota composition and this helminth species, separate models were used which included only this helminth as an explanatory variable. In order to reduce the complexity of fitted models, we did not consider sex as a confounding variable, as it was shown to have a negligible effect on gut microbiota in the study population in preliminary analyses (data not shown). All statistical analyses were performed in R v. 3.1.0 (R Core Team 2014).

### (e) Helminth–microbiota interactions: diversity

Preliminary analyses showed that the number of observed OTUs was a good proxy for microbiota alpha diversity. To assess whether there was an association between microbiota diversity, helminths within their gut niche (the location(s) of the gut in which the helminths reside) and sections of the gut microbiota sampled, we used linear mixed effect models (LMEs) with microbiota diversity as a response variable and presence or abundance of helminths, gut section and helminth-gut section interactions as explanatory variables. Log-transformed read counts for each gut section within each host were used as covariates to account for the fact that the probability of OTU detection varied with sequencing depth. The effects of individual mouse and geographical location were modelled as a nested random intercept. Backwards stepwise deletion of non-significant terms was used to produce the most parsimonious model.

### (f) Helminth–microbiota interactions: composition

To determine whether there were differences in microbiota composition associated with helminth colonization we used distance-based redundancy analysis (db-RDA; capscale function in R package *vegan*) at the whole-gut level, within gut sections and in relation to helminth diversity (i.e. number of helminth species detected). Ecological distances between helminth-associated microbiota communities were assessed using Bray–Curtis dissimilarities (i.e. compositional dissimilarity index that accounts for proportional differences of OTUs among samples) and weighted UniFrac distances that account both for proportional differences of OTUs and their phylogenetic relatedness [50]. OTU tables were randomly rarefied before calculation of dissimilarity matrices to achieve an even sequencing depth corresponding to a minimal number of reads per sample in gut sections that were included in a given analysis. This approach was applied in order to maximize statistical power in corresponding analyses. Geographical location was included as a conditional variable to account for its potentially confounding effect. Significance was assessed using permutation-based marginal tests.

### (g) Helminth–microbiota interactions: OTU abundance

To determine how OTUs varied with helminth infection, we first identified OTUs with a differential abundance (i.e. number of reads corrected for sequencing depth) that varied with helminth presence and abundance in each gut section, using an approach based on generalized linear models with negative binomial errors implemented in the DESeq2 package [51]. These analyses were run using the default pipeline set-up in DESeq2, and significance values were derived using likelihood-ratio tests.

To determine how the relative proportion of OTUs varied with helminth infection, we used the proportion of reads corresponding to OTUs that varied significantly (obtained from the DESeq2 models) as a response variable in log-linear LMEs. We considered log 2-fold changes (calculated using DESeq2, after Anders & Huber [51]) in the proportion of OTUs in any gut section-helminth species combination as an ‘effect size’ of OTU variation. To determine how the proportion of helminth-associated OTUs clustered according to gut section and helminth species combination (i.e. five gut sections for each of the three helminth species), we produced a bootstrapped dendogram, using the package *pvclust*. Furthermore, we created a heatmap based on log 2-fold changes in read counts for OTUs that were significantly associated with helminths. OTUs in the heatmap were clustered based on euclidean distances and a ‘ward’ algorithm to visually highlight groups of OTUs that have a different response between helminths and/or gut sections. The optimal number of OTU clusters was identified using Mantel correlations between the original distance matrix and the binary matrices calculated using dendrogram cuts.

### (h) Helminth-associated variation in the predicted metagenome

To identify metagenomic features in each gut section that were associated with the presence and abundance of particular helminths, we used COG categories as a response variable in DESeq2 analyses.

## 3. Results

**Figure 1.**
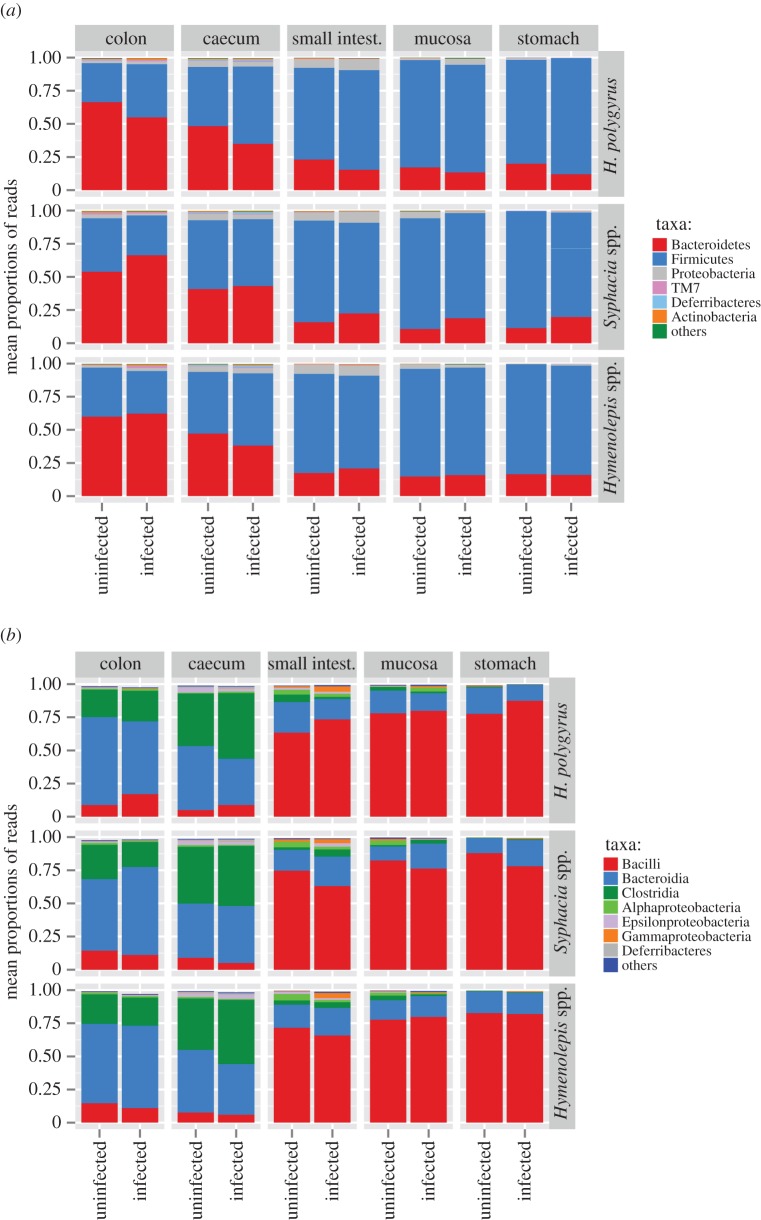
Mean proportions of reads of bacterial (*a*) phyla and (*b*) classes in 29 *A. flavicollis* infected and uninfected by the three most common helminths (*Hymenolepis* spp., *Syphacia* spp. and *H. polygyrus*).

Within the gastrointestinal tract of 29 *A. flavicollis,* two helminth species (*T. muris* and *Aspiculuris tetraptera*) were present in only one individual, so these helminths were excluded from further analyses (table 1). Instead, two nematodes, *H. polygyrus* and *Syphacia* spp., and cestodes *Hymenolepis* spp. were ubiquitous in each of three rodent populations sampled. In addition, *M. muris* was found in only one population. Each helminth colonized distinct parts of the gut; *Hymenolepis* spp*.* and *H. polygyrus* were detected exclusively in the small intestine, *M. muris* in the stomach and *Syphacia* spp*.* in the caecum and to a lesser extent in the colon. Transmission routes to the host for each of the helminth species is via ingestion, although life cycles differ between species such that *Hymenolepis* spp. and *M. muris* are acquired via ingestion of an insect intermediate host while *H. polygyrus* is acquired as infectious larvae and *Syphacia* spp. as infectious eggs. The majority of the sampled rodents were infected with at least one helminth species (93% prevalence) with over half infected with two or more helminth species (57% prevalence). The abundance of *H. polygyrus* and *Hymenolepis* spp. were comparable (mean ± s.e.: 2.79 ± 1.01 and 5.62 ± 1.61), but much lower than that of *Syphacia* spp. (51.69 ± 35.18; table 1). Of the 29 individuals, we did not analyse the microbiota of 10 stomach, two mucosa, one small intestine and one caecum samples owing to a low quantity of DNA template and/or PCR products. In brief, overall the gut microbiota was dominated by Firmicutes (67% of reads) and Bacteroidetes (27%), while Proteobacteria represented 4% and other bacterial phyla by more than 1%. The stomach, small intestine and mucosa were dominated by members of the class Bacilli (78% of reads), whereas the colon and caecum contained more Bacteroidia (49%) and Clostridia (34%; figure 1).

**Table 1.** Summary of mean abundance and prevalence of all helminths infecting *A. flavicollis* at three geographical locations.

		all sample locations					
helminth	helminth niche	prevalence	load (mean)	load (s.e.)	Cadine prevalence	Covelo Prevalence	Pietramurata prevalence
*Syphacia* spp.	caecum	0.59	51.69	35.18	0.63	0.43	0.64
*H. polygyrus*	small intestine	0.45	2.76	1.01	0.63	0.43	0.36
*M. muris*	stomach	0.14	0.55	0.32	0.00	0.00	0.29
*T. muris*	caecum	0.03	0.03	0.03	0.00	0.14	0.00
*A. tetraptera*	colon	0.03	0.34	0.34	0.00	0.00	0.07
*Hymenolepis* spp.	small intestine	0.59	5.62	1.61	0.75	1.00	0.29

### (a) Helminth–microbiota interactions: diversity

Associations between gut microbiota diversity and helminth presence, abundance (the latter detailed in the electronic supplementary material) or diversity were not found at the whole-gut level, or between gut sections (*p* > 0.05 in all cases). The microbiota diversity did, however, differ significantly between gut sections (Δd.f. = 4, *χ*^2^ = 162.090, *p* < 0.001).

### (b) Helminth–microbiota interactions: composition

**Figure 2.**
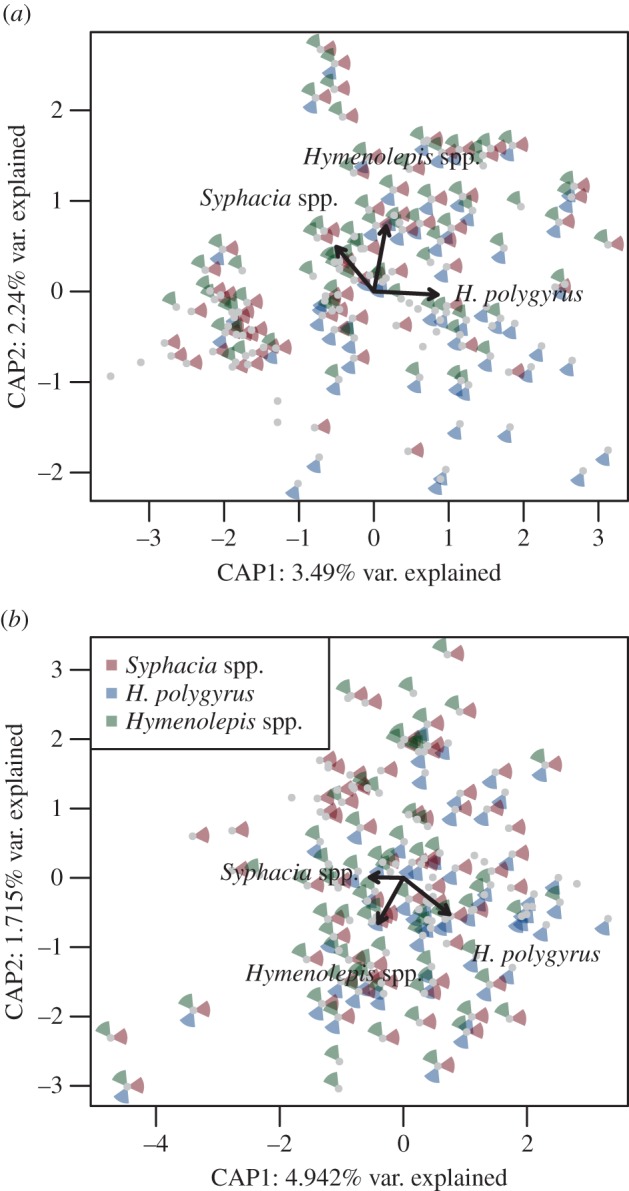
Ordination plots for the overall association between gut microbiota content variation and the presence of three common helminths using (*a*) Bray–Curtis and (*b*) weighted UniFrac dissimilarities as the response variable (both analyses are controlled for variability in gut microbiota between different gut sections and geographical locations). Distribution of samples along the first two db-RDA axes (i.e. CAP1 and CAP2) and associated proportion of variation are shown. The presence of individual helminths is indicated by the coloured segments surrounding the data points (see the figure key). The length of the arrow indicates the relative importance of each helminth; bold arrows indicate a significant effect (all partial effects of individual helminths were significant; permutation-based *p* < 0.05).

Taxonomic assignment of OTUs to phylum and class level did not reveal any pronounced changes in community composition whether helminths were present or absent (figure 1). However, *H. polygyrus* presence was associated with a slight increase in the Firmicutes/Bacteroidetes ratio along the whole gut for infected individuals (mean ± s.e.: 23.151 ± 28.894 and 50.600 ± 10.398), although this effect was not significant (LME: Δd.f. = 1, *χ*^2^ = 2.747, *p* = 0.0974). Instead, constrained ordination (db-RDA) revealed the presence of any of the three common helminths to be associated with significant changes in whole-gut bacterial communities (table 2 and figure 2), although the effect size was low. Instead, *Syphacia* spp. are associated with a gut microbiota that is divergent, in terms of composition, to that associated with *H. polygyrus* (figure 2). By contrast, the community composition associated with *Hymenolepis* spp. was not associated with either of the other two common helminths, and these differences were consistent regardless of which distance index was used (figure 2). The variance, however, explained in the helminth-associated community composition was very low (adjusted *R*^2^ ranged between 0.004 and 0.016; table 2). Helminth abundance was also associated with significant changes in the overall microbiota composition, except for *Hymenolepis* spp. which was marginally non-significant (*p* = 0.079), although, again, the proportion of variance explained was still low (adjusted *R*^2^ ranged between 0.002 and 0.014; table 2)

**Table 2.** Association between the (I) presence and (II) abundance of common helminths (*Syphacia* spp.*, H. polygyrus, Hymenolepis* spp.) and (A) taxonomic and (B) predicted functional gut microbiota composition. Models were fitted for samples from all gut sections (whole intestine) and subsequently separately for each gut section. Values of pseudo-*F* statistics, associated degrees of freedom, permutation-based *p*-values and proportion of variance explained (adjusted *R*^2^) are given. Significant effects (*p* < 0.05) are in bold type.

				whole intestine			colon (*n* = 29)			caecum (*n* = 28)		
type of explanatory variables	response variable	distance metric	helminth	*F*	*p*	adj. *R*^2^	*F*	*p*	adj. *R*^2^	*F*	*p*	adj. *R*^2^
(I) helminth presence	(A) OTUs	Bray–Curtis	*Hymenolepis* spp.	**2.794**	**0.005**	**0.016**	0.810	0.850	−0.007	1.014	0.500	0.001
			*Syphacia* spp.	**2.349**	**0.005**	**0.009**	1.297	0.077	0.012	0.988	0.500	0.000
			*H. polygyrus*	**3.513**	**0.005**	**0.012**	0.887	0.740	−0.004	1.163	0.300	0.007
		weighted UniFrac	*Hymenolepis* spp.	**2.980**	**0.010**	**0.013**	0.450	0.950	−0.021	0.538	0.910	−0.017
			*Syphacia* spp.	**1.820**	**0.049**	**0.004**	1.267	0.230	0.011	0.490	0.880	−0.019
			*H. polygyrus*	**3.986**	**0.010**	**0.009**	1.735	0.110	0.029	**2.006**	**0.035**	**0.041**
	(B) COG categories	Bray–Curtis	*Hymenolepis* spp.	**4.070**	**0.005**	**0.015**	1.366	0.260	0.016	1.589	0.170	0.023
			*Syphacia* spp.	0.979	0.320	0.001	2.690	0.100	0.067	1.236	0.260	0.011
			*H. polygyrus*	**4.339**	**0.005**	**0.016**	2.032	0.150	0.042	**4.779**	**0.030**	**0.134**
(II) helminth abundance	(A) OTUs	Bray–Curtis	*Hymenolepis* spp.	**2.367**	**0.005**	**0.018**	0.844	0.720	−0.006	0.974	0.720	−0.001
			*Syphacia* spp.	**3.069**	**0.005**	**0.014**	1.354	0.058	0.014	1.234	0.150	0.009
			*H. polygyrus*	**3.741**	**0.005**	**0.009**	0.958	0.610	−0.002	1.338	0.061	0.014
		weighted UniFrac	*Hymenolepis* spp.	1.802	0.079	0.011	0.605	0.750	−0.015	0.670	0.760	−0.012
			*Syphacia* spp.	**2.100**	**0.023**	**0.005**	1.373	0.240	0.015	0.621	0.780	−0.014
			*H. polygyrus*	**3.486**	**0.010**	**0.004**	1.479	0.190	0.020	**1.962**	**0.049**	**0.039**
	(B) COG categories	Bray–Curtis	*Hymenolepis* spp.	1.641	0.125	0.004	0.450	0.540	−0.022	0.838	0.430	−0.003
			*Syphacia* spp.	1.101	0.360	0.002	1.708	0.200	0.033	0.885	0.330	−0.002
			*H. polygyrus*	**3.723**	**0.015**	**0.014**	1.394	0.290	0.019	**4.912**	**0.018**	**0.139**

				small intestine (*n* = 28)			mucosa (*n* = 27)			stomach (*n* = 19)		
type of explanatory variables	response variable	distance metrics	helminth	*F*	*p*	adj. *R*^2^	*F*	*p*	adj. *R*^2^	*F*	*p*	adj. *R*^2^
(I) helminth presence	(A) OTUs	Bray–Curtis	*Hymenolepis* spp.	1.306	0.260	0.013	0.984	0.360	0.001	1.870	0.120	0.062
			*Syphacia* spp.	0.615	0.760	−0.014	1.075	0.340	0.005	0.644	0.600	−0.023
			*H. polygyrus*	1.914	0.062	0.037	**2.617**	**0.040**	**0.067**	0.472	0.730	−0.035
		weighted UniFrac	*Hymenolepis* spp.	1.425	0.130	0.021	1.327	0.320	0.020	**5.771**	**0.013**	**0.240**
			*Syphacia* spp.	0.585	0.770	−0.015	1.204	0.210	0.014	1.325	0.280	0.022
			*H. polygyrus*	1.698	0.110	0.033	1.848	0.150	0.042	2.258	0.145	0.067
	(B) COG categories	Bray–Curtis	*Hymenolepis* spp.	1.301	0.280	0.016	0.925	0.410	0.001	**5.306**	**0.023**	**0.211**
			*Syphacia* spp.	0.301	0.770	−0.027	0.689	0.500	−0.010	0.787	0.480	−0.007
			*H. polygyrus*	1.608	0.180	0.030	0.649	0.580	−0.012	2.065	0.155	0.055
(II) helminth abundance	(A) OTUs	Bray–Curtis	*Hymenolepis* spp.	0.804	0.550	−0.007	0.620	0.750	−0.014	1.678	0.170	0.049
			*Syphacia* spp.	1.131	0.330	0.006	1.911	0.210	0.038	0.773	0.550	−0.014
			*H. polygyrus*	1.724	0.100	0.030	**2.934**	**0.010**	**0.079**	0.600	0.660	−0.026
		weighted UniFrac	*Hymenolepis* spp.	0.442	0.810	−0.022	0.574	0.650	−0.013	**5.282**	**0.013**	**0.232**
			*Syphacia* spp.	1.300	0.200	0.016	1.948	0.103	0.047	0.485	0.740	−0.022
			*H. polygyrus*	1.333	0.300	0.018	1.900	0.140	0.045	1.523	0.170	0.033
	(B) COG categories	Bray–Curtis	*Hymenolepis* spp.	0.535	0.690	−0.018	0.685	0.590	−0.010	**4.684**	**0.017**	**0.191**
			*Syphacia* spp.	0.740	0.470	−0.009	0.937	0.430	0.002	0.344	0.800	−0.030
			*H. polygyrus*	0.843	0.500	−0.004	0.724	0.450	−0.008	1.449	0.230	0.026

Interestingly, at the gut-section level, significant changes were not always co-localized within the gut niche of a given helminth species. In addition, significance varied according to the distance index used, probably as a result of the generally low effect size of observed changes (table 2). However, our results do not indicate any difference in the discriminatory power of the two distance indexes used, i.e. the effect of helminth presence in individual gut sections had significant effect in two cases based on weighted UniFrac and in one case based on Bray–Curtis dissimilarities. The same was true for abundance-based analyses. *Hymenolepis* spp., normally found colonizing the small intestine, was associated with significant community composition changes in the stomach (based on weighted UniFrac distances; table 2). *H. polygyrus,* found in the small intestine mucosa, was associated both with significant changes in the microbiota of the mucosa (based on Bray–Curtis) and in the caecum (based on weighted UniFrac), but not in the lumen of the small intestine itself (table 2). No association was found between helminths and the microbiota composition of the small intestine and colon, although *Syphacia* spp., usually found in the caecum and to a lesser extent in the colon, was associated with marginally non-significant microbiota composition changes in the colon, but not in the caecum (table 2). However, the proportion of variance explained by the presence of helminths was relatively low for all gut sections (range 0.03–0.13, table 2), except for the effect of *Hymenolepis* spp. on the stomach gut microbiota (adjusted *R*^2^ = 0.24, table 2). Using helminth abundances as the explanatory variable provided the same patterns as above (table 2).

*M. muris* was associated with significant changes in the gut microbiota composition at the whole-gut level (Bray–Curtis and weighted UniFrac distances: pseudo-*F*_1,60_ = 3.634, *p* = 0.005, adjusted *R*^2^ = 0.036 and pseudo-*F*_1,128_ = 5.015, *p* = 0.005 adjusted *R*^2^ = 0.033), but not for individual gut sections (*p* > 0.15 in all cases). *M. muris* abundance also altered whole-gut microbiota composition (*F*_1,60_ = 3.488, *p* = 0.005, adjusted *R*^2^ = 0.034 and *F*_1,60_ = 4.114, *p* = 0.005, adjusted *R*^2^ = 0.026), but not at the gut section level (*p* > 0.100 in all cases).

A significant association between the helminth diversity and gut microbiota composition was found based on Bray–Curtis dissimilarities (*F*_1,123_ = 2.584, *p* = 0.005), but not with weighted UniFrac distances (*F*_1,123_ = 1.552, *p* = 0.170). The associated proportion of variance explained was negligible in both cases (adjusted *R*^2^ = 0.011 and 0.003, respectively).

### (c) Helminth–microbiota interactions: OTU abundance

The proportion of OTUs significantly associated with each of the three common helminths across the gut was low (DESeq2: 239 OTUs in total, 3.7% of all OTUs). Similarly, the proportion of OTUs affected by any given helminth was also low at the gut section level, ranging between 0% and 5%. The number of OTUs affected by helminth abundance was lower than number of OTUs affected by helminth presence (DESeq2: 185 OTUs in total, 2.6% of all OTUs) as was the estimated proportion of microbiota affected by helminth abundance (0–2.2%) and presence (0–5%).

**Figure 3.**
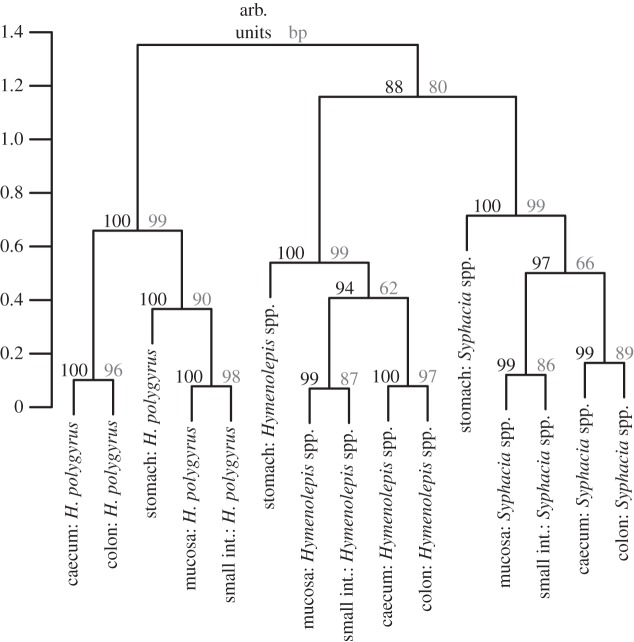
Bootstrapped hierarchical clustering of log 2-fold change vectors for three common helminths (*Syphacia* spp., *H. polygyrus* and *Hymenolepis* spp.) in five gut sections. Log 2-fold change vectors characterize gut microbiota modulation induced by the presence of a particular helminth in different gut sections. Proximity in the dendrogram indicates a similar response and the scale of *y*-axis corresponds to distances among log 2-fold change vectors. Approximate unbiased (arb. units, in black) and bootstrap probability values (bp, in grey) are reported above individual nodes.

**Figure 4.**
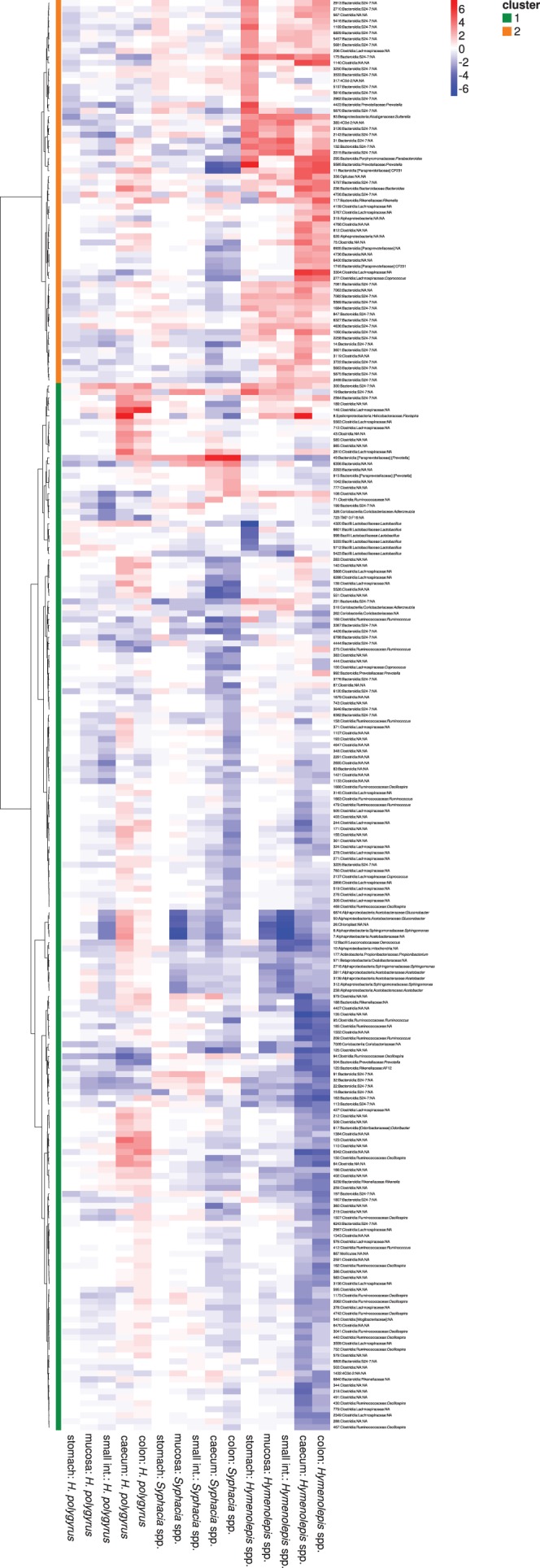
Heatmap of log 2-fold changes of OTUs that were significantly associated with the presence of at least one of the three common helminths (*Syphacia* spp., *H. polygyrus* and *Hymenolepis* spp.) in at least one gut section after DESeq2 analyses. Negative (blue) and positive (red) values indicate a decrease or increase, respectively, of a given OTU owing to the presence of a given helminth. Dendrogram on left-hand side: OTUs were grouped in two clusters according to Euclidean distances between associated log 2-fold changes and a Ward algorithm (see main text for more details). OTU identifications and their taxonomic assignations are listed on the right-hand side.

Hierarchical clustering of gut microbiota changes indicated that common helminths modulate the gut microbiota in distinct ways irrespective of the gut section, each forming separate, highly supported clusters (bootstrap support = 80–99, approximate unbiased *p* values = 88–100; figure 3). In addition, the clustering patterns were consistent, such that the microbiota changes induced by the presence of any helminth in the caecum and colon were always highly correlated, as were the changes in the mucosa and small intestine, with the stomach distinct from all other gut sections (figure 3). Analyses of helminth abundance revealed the same pattern (detailed in the electronic supplementary material). Consistent with this clustering, marked variation in gut microbiota changes owing to the presence of different helminths in different gut sections was also evident from the heatmap (figure 4; electronic supplementary material, figure S1). The OTUs clustered in two distinct groups: the first was dominated by Lachnospiraceae, Lactobacillaceae, Ruminococcaceae, Acetobacteraceae, Sphingomonadaceae and within the Bacteroidetes the S24–7 family, which were underrepresented in all gut sections of hosts infected by *Hymenolepis* spp. and *Syphacia* spp*.,* whereas their response to *H. polygyrus* was neither under- or overrepresented (figure 4). The second cluster was dominated by S24–7, and corresponding OTUs were overrepresented in hosts infected by *Hymenolepis* spp., whereas infection by *S. obvelata* and *H. polygyrus* were associated with a decrease or no abundance change of these OTUs in most cases (figure 4)*.* OTUs associated with helminth abundance resulted in four separate groups based on hierarchical clustering; nevertheless, similar to cluster two in the analyses of helminth presence, a group of OTUs dominated by S24–7 and positively correlated with *Hymenolepis* spp. was still evident (see the electronic supplementary material). OTUs negatively affected by *M. muris* presence and abundance corresponded predominantly to S24–7, whereas those positively affected were variable and differed between gut sections.

### (d) Helminth-associated variation in the predicted metagenome

**Figure 5.**
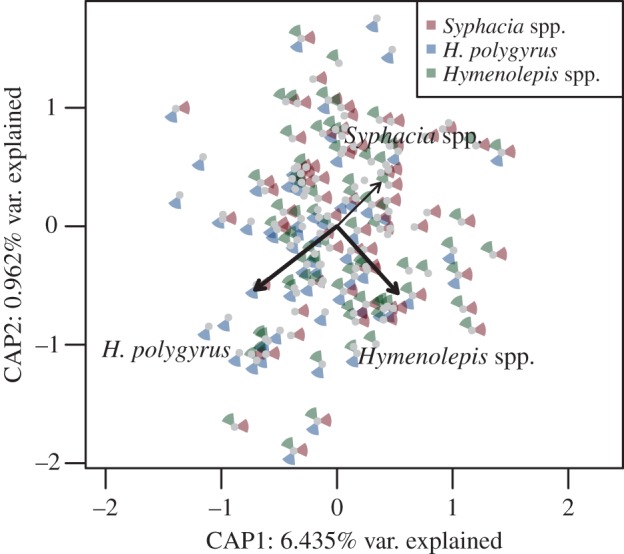
Ordination plots of the association between predicted metagenomic content and helminth presence, based on Bray–Curtis dissimilarities. Distribution of samples along the first two db-RDA axes (i.e. CAP1 and CAP2) and associated proportion of variation are shown. The presence of individual helminths is indicated by coloured segments surrounding the data points (see the figure key). Significant effects of the helminths are indicated by bold arrows (permutation-based *p* < 0.05).

A global model using constrained ordination (db-RDA) of the entire gut revealed a significant effect on the predicted metagenome composition defined by COG categories owing to the presence of *H. polygyrus* and *Hymenolepis* spp., although the proportion of variance explained was low (adjusted *R*^2^ = 0.019 and 0.015, figure 5 and table 2). At a gut-niche level, *H. polygyrus* and *Hymenolepis* spp. presence were significantly associated with the functional variation of the caecal and stomach metagenomes, respectively, but *Syphacia.* spp. showed no association with any gut section (table 2). We also detected a significant effect of *H. polygyrus* abundance across the whole gut, but not for *Hymenolepis* spp. and *Syphacia* spp*.* abundance (table 2).

*Hymenolepis* spp. presence was associated with variation in bacterial taxa from several COG categories in the stomach, but not in other gut sections. Two COG categories related to prokaryote virulence—[U] intracellular trafficking, secretion, and vesicular transport and [M] cell wall/membrane/envelope biogenesis—increased, while another, [F] nucleotide transport and metabolism, as well as [S], of unknown function, decreased owing to *Hymenolepis* spp. presence. Analyses for abundance suggested that a higher number of COG categories in the stomach metagenome were associated with *Hymenolepis* spp. (see the electronic supplementary material). We did not find any effect of *Syphacia* spp. and *H. polygyrus* presence on the predicted abundance of COG categories in any gut section. On the contrary, *H. polygyrus* abundance was associated with an increase of [G], carbohydrate transport and metabolism in the caecum. At a whole-gut level, *M. muris* presence was associated with significant variation in the gut metagenome (*F*_1,60_ = 4.441, *p* = 0.017 adjusted *R*^2^ = 0.035). At a gut-niche level, a marginally insignificant effect of *M. muris* presence was found on the small intestine metagenome (*F*_1,11_ = 3.237, *p* = 0.0580, adjusted *R*^2^ = 0.165), but little effect at the gut-section level, also for abundance (*p* > 0.2 in all cases). No COG category was significantly associated with *M. muris* presence or abundance.

## 4. Discussion

In this study, we examined the association between multiple helminth infections and microbiota diversity and composition within and between multiple gut sections of three populations of wild mice. We found that three common helminths (*H. polygyrus*, *Syphacia* spp. and *Hymenolepis* spp.) were not associated with changes in gut microbiota diversity, but they did alter the composition of these microbial communities. In general, evidence for helminth-associated change in microbiota diversity and OTU abundance in the literature is mixed. In laboratory animals, the effect of nematodes has been shown to be relatively large; for example, C57BL/6 mice experimentally infected with *H. p. bakeri* resulted in significant shifts in the composition of gut microbiota, which almost doubled in abundance in the ileum (helminth's niche), but not in the caecum [10]. By contrast, studies on domesticated pigs challenged with *T. suis* did not reveal any change in bacterial diversity compared with naive uninfected pigs [24,26]. We may expect that large changes in microbiota diversity or taxa abundance seen in some studies may be a dose-dependent effect owing to the high inoculum typically given in laboratory studies: 200 larvae in mice studies [10,20] and 20 000 eggs in pigs [26], and in laboratory mice with bacteria abundance two to three times higher when infection loads were high [20].

The majority of animals sampled in the current study were infected with any given helminth (93% prevalence); therefore, infection was the norm. Previous studies suggest that natural helminth infection maintains microbiota diversity, and experimental inoculation in parasite-free hosts restores it, suggesting a capacity of helminths to maintain a high gut species richness [18,23]; as such, colonization by helminths may represent gut homeostasis. Evidence of helminth-maintained microbiota diversity has been noted elsewhere; for example, analyses of faecal microbiota in an indigenous community in Malaysia compared naturally parasite-free versus those naturally infected, finding a higher microbiota diversity in those with helminths [23]. It is important to consider that whether sustained loss of helminths results in a loss of microbiota diversity and an increase in disease, the relative importance of this association in relation to other drivers of diversity loss, such as diet and antibiotic use, must be considered seriously.

At a whole-gut level in our study, the ecological distance between each community suggested that *Syphacia* spp*.* was associated with a gut microbiota that is opposite in terms of community composition to that of *H. polygyrus*, whereas the microbiota composition associated with *Hymenolepis* spp. was not associated with either of the other two common helminths (figure 2); however, the effect size of these changes was very low. In addition, the proportion of OTUs involved in interactions with each of the three dominating helminths was low irrespective of the gut section (0–5%), and constituted only 4% (*n* = 239) of all OTUs. Associations were species-specific, each helminth being associated with selective microbiota modulation. We also carried out a community-level approach that not only observed co-localized associations, but, interestingly, in two cases helminth species were associated with variation in gut microbiota composition up- or downstream from their usual niche, providing evidence of not only local, but also distant effects on gut microbiota. This non-localized effect has been noted by other studies with distinct changes observed in the caecum and colon outside of the niche of *H. p. bakeri,* suggesting a clear role for changes to occur throughout the gut [20]. Consistent with the whole-gut level analyses, our effect sizes were generally low. *Hymenolepis* spp. (normally found in the small intestine) showed the strongest association with microbiota composition, with downstream effects on the caecum and colon, and upstream ones on the stomach microbiota. Interestingly, *Hymenolepis* spp. was also the most prevalent and the largest of the helminth species observed, reaching up to 10 cm in length and occupying the entire small intestine in infected animals. Although the size of the helminth is not necessarily associated with its antigenic ‘strength’, its size may substantially alter the local environment via intake of substances, excretions and secretions or by interacting with host cells and the immune system [52]. We did not, however, observe any additional effects associated with the burden of helminths suggesting the number of helminths are not important in terms of microbiota variation.

Previous studies have shown that helminth-associated changes in microbial community structure and the bacterial taxa interacting with helminths vary considerably. Although such differences could be partly ascribed to the diverse effects of various helminth species, even studies concerning closely related helminths report contradictory findings. For example, therapeutic infection of *T. trichiura* induced massive increases in *Tenericutes* and a decrease in Cyanobacteria (genus *Streptophyta*) in rhesus monkeys suffering from chronic diarrhoea [18], whereas the same helminth species did not induce any detectable changes in the microbiota of healthy humans [22]; however, these results were based on pinch biopsies of the colon and faecal microbiota, respectively. Pronounced microbiota changes were also observed in the colon microbiota of healthy domestic pigs after infection by the related species, *T. suis* [24,26]; however, in this case, different phyla and genera were affected, namely Proteobacteria (genera *Succinivibrio, Desulfovibrio*), Deferribacteres, Spirochaetes (*Spirochaeta*) and Bacteroidetes (*ParaPrevotella*). Likewise, following infection by *H. p. bakeri* of two different inbred laboratory mouse strains, an increase of Lactobacillaceae in the small intestine was observed in C57BL/6 mice [10,20,25], but not in BALB/c [25]. The fact that these changes were most pronounced in small intestine compared with distal gut section is worth noting. The variation observed in studies on mouse models indicates that even slight differences in host genetic background and/or in baseline microbiota composition may have pronounced effects on the outcome of helminth–microbiota interactions. Given this high heterogeneity in previously published observations, it is difficult to assess whether such variation is related to helminth-, microbiota-, gut section- and/or host-specific effects or variation deriving from incongruences in methodologies. However, this knowledge is crucial from a biomedical point of view.

To our knowledge, this is the first study showing that the abundance of microbial taxa varies according to the helminth species colonizing the host gut. Our data suggest that *H. polygyrus* and *Syphacia* spp. have divergent effects on some members of gut microbiota. In particular, an increase in the abundance of several Lachnospiraceae OTUs were associated with *H. polygyrus* infection, whereas *Syphacia* spp. infection was associated with a decrease in the same taxa. In addition, *Syphacia* spp. abundances were correlated with a decrease of several Firmicutes (*Lactobacillus*) OTUs, whereas the opposite (but non-significant) change was observed with *H. polygyrus* infection. The most striking pattern was an increase in the unidentified bacteria, S24–7 OTUs (a member of the phylum of Bacteroidetes with potential effects on host health; [53]) in mice infected by the cestode *Hymenolepis* spp. In addition, *Hymenolepis* spp. affected the abundance of other bacterial genera that may have significant effects on host physiology and health status, such as *Suterella* [54,55], *Sphingomonas* [56,57] and *Flexispora* [58] (figure 4). Overall, the change in predicted metagenomes was low (figure 5), with the proportion of inter-individual variation (both in the composition of microbiota and predicted metagenomes) explained by helminth infection < 5% for each of the three common helminths. Nevertheless, small increases in specific bacterial taxa may influence the production of important metabolites acting on gut homeostasis and influencing both vertebrate and invertebrate host physiology [52].

In conclusion, we find microbiota variation associated with helminths to be species-specific and not confined to the helminth niche as such; therefore, we propose future studies should be approached at a whole-gut and helminth community level and, where possible, in natural populations. Even though the changes we observed in gut microbiota were, in many cases small, it is not clear what the outcomes of this are in the long- or short-term for the host. The question of how much change is significant is hard to answer. In humans, projects such as the Human Microbiome Project (HMP) have endeavoured to understand the compositional and functional states of the gut microbiota so that this can be used as a baseline for understanding dysbiosis, the effects of anthelmintics and antibiotics, but studies in free-living animals are comparatively rare despite their recognized importance [38]. While this investigation has examined the association of helminths with the microbiota, it is important to note that other variables not measured here may play a role: for example, infection may alter metabolites associated with the gut microbial community [59]. Future work should focus on understanding some of the mechanisms playing a role in the three-way interaction between host-microbiota and helminths.

## Supplementary Material

Supplementary material

## Supplementary Material

Supp_Fig_1_aheatmap_SES_2_ward_MULTI_k6_VERY_LARGE_rank.tiff

## Data accessibility

Demultiplexed unfiltered sff files are available at the European Nucleotide Archive: http://www.ebi.ac.uk/ena/data/view/PRJEB8312 (accession numbers: ERS645272–ERS645402).
