# Supplementary material for: Interactions between multiple helminths and the gut microbiota in wild rodents
Source: Philos Trans R Soc Lond B Biol Sci. 2015 Aug 19;370(1675):20140295. doi: 10.1098/rstb.2014.0295 (PMC4528493; doi:10.1098/rstb.2014.0295)

Supplementary material for Kreisinger, J. *et al.* 2015. Interactions between multiple helminths and the gut microbiota in wild rodents. *Phil. Trans. R. Soc. B.* doi: 10.1098/rstb.2014.0295

Interactions between helminth abundance and gut microbiota

The abundance of helminths found in 29 wild *Apodemus flavicollis* were rank-transformed and rescaled from 0 (no infection) to 1 (maximal infection detected). Consequently, the resulting regression coefficients correspond to predicted changes in gut microbiota between uninfected mice and those with the highest abundance.

Helminth-microbiota interactions: Diversity

There were no significant relationships between the overall OTU richness along the gut and the abundance of three common helminths after controlling for the effect of gut location, sex and sequencing depth (estimate ± s.e.: *Hymenolepis* spp: -57.60 ± 61.29, Δ D.f. = 1, χ^2 = 0.86, p = 0.35; *Syphacia* spp.: -93.25 ± 65.13, Δ D.f. = 1, χ^2 = 2.030, p = 0.154; *H. polygyrus*: -17.62 ± 60.41, Δ D.f. = 1, χ^2 = 0.085, p = 0.771), nor were any two-way interactions significant (p > 0.100 in all cases). A non-significant decrease of overall microbiota diversity with *M. muris* abundance was detected (estimate ± s.e.: -117.11 ± 75.00, Δ D.f. = 1, χ^2 = 2.2171, p = 0.1365).

Helminth-microbiota interactions: Composition

Contrasting with the effect of presence alone, the effect of *Hymenolepis* spp. abundance on the whole gut microbiota was marginally non-significant using weighted unifrac distances (db-RDA: p = 0.079). In addition, abundance, but not presence of *H. polygyrus* had a marginally non-significant effect on the caecum microbiota composition based on Bray-Curtis distances (p = 0.061 and p = 0.300 respectively). However, consistent with analyses based on *M. muris* presence, *M. muris* abundance was associated with whole-gut changes of microbiota composition (db-RDA on Bray Curtis and weighted unifrac distances: $F_{(1,60)}$ = 3.488, p = 0.005, adjusted R^2 = 0.034 and $F_{(1,60)}$ = 4.114, p = 0.005 adjusted R^2 = 0.026). As for the *M. muris* presence, we did not detect any effect of *M. muris* abundance on individual gut sections (p > 0.100 in all cases).

Helminth-microbiota interactions: OTU abundance

Gut microbiota variation (assessed as a log 2-fold changes in OTU abundance) due to the presence of each of three common helminths at each gut sections sampled was highly

correlated with microbiota changes due to helminth abundance (Pearson corr. coeffs.: mean = 0.878, S.E. = 0.013, range = 0.780 – 0.942).

OTUs in the heatmap produced four distinct clusters (supplementary Figure 1), instead of two (Fig. 4, main text). Similar to helminth presence, one cluster was dominated by S24-7 OTUs and the abundance of these OTUs increased with that of *Hymenolepis* spp. (supplementary Figure 1, cluster number 3). In addition, *Lactobacillus* OTUs from cluster number 3 were positively associated with *H. polygyrus* abundance, but negatively associated with *Syphacia* spp. abundance. The opposite pattern was observed for one *Prevotella* OTU (cluster number 2). The number of OTUs affected by *M. muris* abundance was higher for the caecum and colon (n = 73 and 114) compared to the small intestine, small intestine mucosa and stomach (n = 23, 16 and 9), consistent with *DESeq2* analyses for *M. muris* presence.

Helminth-associated variation in the predicted metagenome

Results of the metagenomic predictions and helminth abundance were consistent with helminth presence (Table 2, main text) with one exception: we did not detect a significant effect of *Hymenolepis* spp. abundance on the whole gut variation of predicted metagenomes ($F_{(1,121)} = 1.641$, $p = 0.125$, adjusted $R^2 = 0.004$). As was the case for presence, *M. muris* abundance indicated a slight effect on whole gut metagenome variation ($F_{(1,60)} = 3.173$, $p = 0.023$, adjusted $R^2 = 0.023$). However at the individual gut sections no effect of variation was found ($p > 0.100$ in all cases, adjusted R^2 range = -0.063 – 0.135), including the small intestine metagenomes ($F_{(1,11)} = 2.759$, $p = 0.130$, adjusted $R^2 = 0.135$).

Contrary to the results of helminth presence, *DESeq2* analyses for helminth abundance indicated that *H. polygyrus* was associated with an increase of COG category [G] Carbohydrate transport and metabolism in the caecum. However, similar to helminth presence, *Hymenolepis* spp. abundance was associated with an increase of [U] and [M] and a decrease of [F] and [S] in the stomach, but not in other gut sections. In addition, unlike *DESeq* analyses for the effect of *Hymenolepis spp.* presence on the stomach metagenome, *Hymenolepis spp.* loads were positively linked with an increase of [A] RNA processing and modification and [C] Energy production and conversion and negatively associated with [D] Cell cycle control, cell division and [K] Transcription. Consistent with analyses on helminth presence, we did not detect any COG categories to be associated with *Syphacia* spp. or *M. muris* abundance in any gut section.

Supplementary Figure 1: Heatmap for log 2-fold changes of OTUs that were significantly associated with infection load of at least one of the three helminths (Syphacia spp., H. polygyrus, Hymenolepis spp.) in at least one gut section based on DESeq2 analyses. Negative (blue) and positive (red) values indicate decrease and increase of a given OTU due to presence of a particular helminth. OTUs were grouped into four clusters according to euclidean distances between associated log 2-fold changes and a ward algorithm.

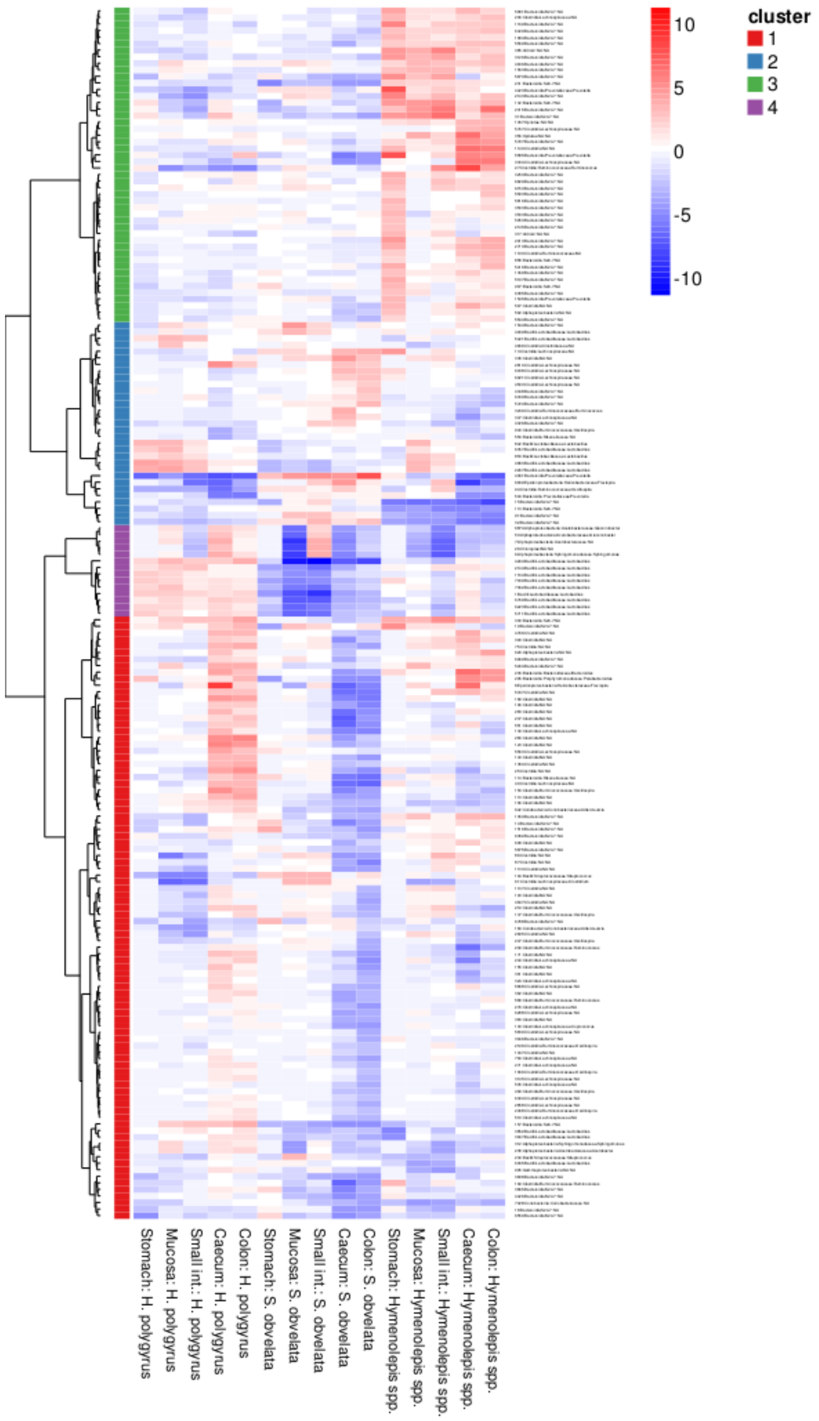

Supplement: Supplementary material [file rstb20140295supp1.pdf]
