# Supplementary figures and images for: Interactions between multiple helminths and the gut microbiota in wild rodents
Source: Philos Trans R Soc Lond B Biol Sci. 2015 Aug 19;370(1675):20140295. doi: 10.1098/rstb.2014.0295 (PMC4528493; doi:10.1098/rstb.2014.0295)

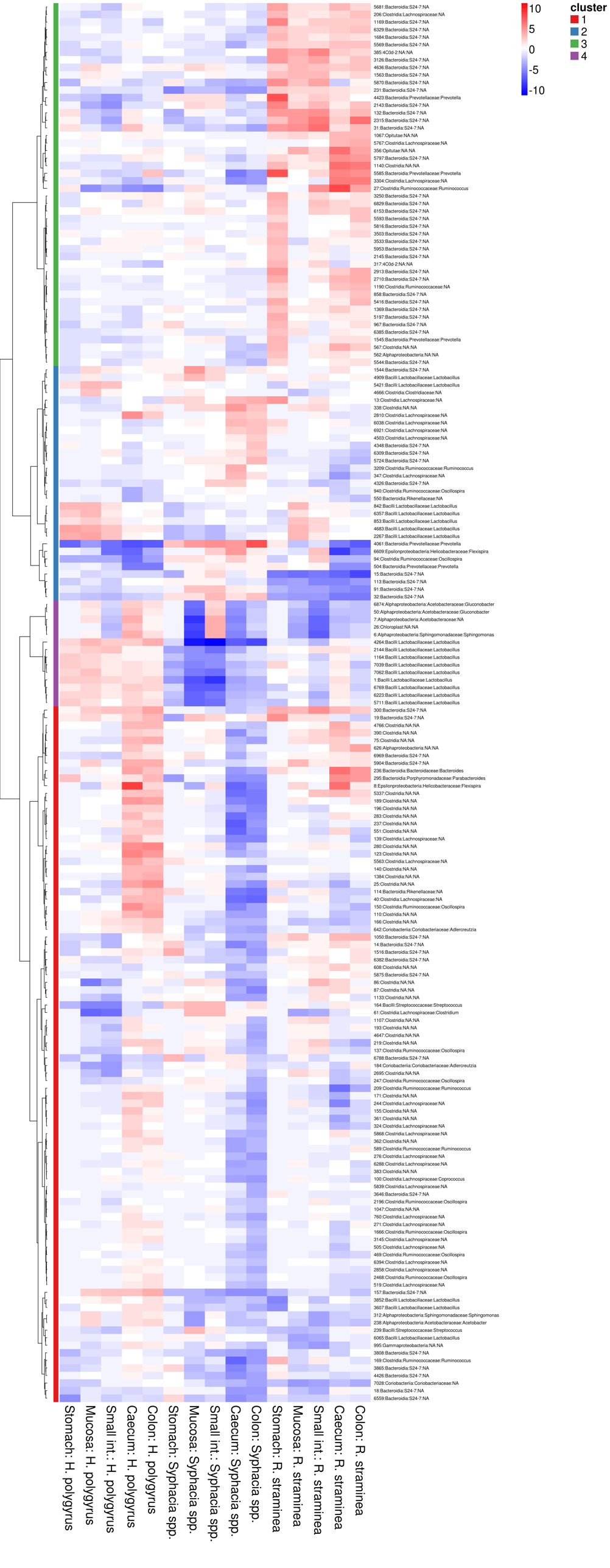

Supplement: Supp_Fig_1_aheatmap_SES_2_ward_MULTI_k6_VERY_LARGE_rank.tiff [file rstb20140295supp2.tiff]
